# Case Report: The Application of Dupilumab in Atopic Dermatitis Children Complicated With Nephrotic Syndrome

**DOI:** 10.3389/fmed.2022.813313

**Published:** 2022-04-29

**Authors:** Ya-Qi Yang, Hao Chen, Li-Ru Qiu, Rong-Fei Zhu

**Affiliations:** ^1^Department of Allergy, Tongji Hospital, Tongji Medical College, Huazhong University of Science and Technology, Wuhan, China; ^2^Department of Pediatrics, Tongji Hospital, Tongji Medical College, Huazhong University of Science and Technology, Wuhan, China

**Keywords:** case report, Dupilumab, atopic dermatitis, nephrotic syndrome, efficacy and safety

## Abstract

Nephrotic syndrome (NS) tends to be more common in patients with history of allergies. Atopic dermatitis (AD) is one of the most common allergic diseases in children. Dupilumab, a dual IL-4 and IL-13 inhibitor, has been widely used to treat AD patients. However, the efficacy and safety of Dupilumab in NS is unclear. We reported two AD patients with NS comorbidities treated with Dupilumab. The outcomes showed the good control of NS and less systemic steroids and/or immunosuppressive agents use during the Dupilumab treatment period, accompanied by significant relief of AD symptoms. We suggest prospective pilot studies and randomized controlled trials could be carried out to validate the efficacy and safety of Dupilumab in the treatment of NS patients.

## Introduction

Atopic dermatitis (AD) is one of the most common allergic diseases in children, with a prevalence of more than 20% in high-income countries, which seriously affects the life quality of children (1). In China, the prevalence of AD also reached 14% in the general population and nearly 13% in children (2, 3). Nephrotic syndrome (NS) is a rare pediatric kidney disease, with an average incidence of 2–16.9 per 100,000 children worldwide (4).

There are reports that the risk of nephrotic syndrome in children with AD is seven times higher than those without AD (5). Many studies suggest that the pathogenesis of NS might be correlated to Th2 activation and resulted in a Th1/Th2 imbalance. The activated Th2 cells produce signatured type 2 cytokines such as IL-4 and IL-13, which will promote the synthesis and secretion of immunoglobulin E (IgE) through B cells. This may be one of the mechanisms for the conjecture that NS and allergic diseases are correlated (6, 7). Clinical trials and real-world studies have confirmed the efficacy and safety of Dupilumab (a human monoclonal antibody against interleukin (IL)-4Rα and a dual inhibitor of IL-4 and IL-13 signaling) in the treatment of pediatric AD (8–12). However, it is unclear whether Dupilumab can be used in NS complicated with AD children. We treated patient #1 and patient #2 with Dupilumab at Tongji Hospital, Tongji Medical College of Huazhong University of science and technology, and reviewed the relationship between AD and NS.

## Case Description

The first patient was a 9-year-old boy who suffered from recurrent erythema and papules over body and diagnosed as infant AD after birth. The manifestation of AD included a long history of intense pruritus, dry skin, recurrent erythema and papules, lichenized and excoriated plaques of the skin, mostly appearing on the limbs and affecting flexor surfaces in a symmetrical distribution. His AD symptoms were worsened in summer especially after perspiration. He was given topical glucocorticoids and oral antihistamines irregularly to control the AD symptoms, but didn't respond well to the treatments. He developed allergic rhinitis (AR) when he was 7 years old. His mother also had AR history. When he was 5 years old, he suffered from anasarca and proteinuria (urinary albumin excretion rate >1,500 mg/day) and was diagnosed with NS. He received prednisone 1 mg/kg daily to control the NS comorbidity and gradually tapered to 2.5 mg daily according to urine albumin level. The patient had a height of 137 cm, weight of 32 kg and the body mass index of 17.05 kg/m^2^ when he presented to our department. Investigator's global assessment (IGA; ranging from “0” to “5”, “0” for none, “5” for very severe), the body surface area (BSA) (ranging from “0%” to “100%”) and the eczema area and severity index (EASI) (ranging from “0” to “72”, “0” for none, “72” for very large area involved and very severe) were used to assess the severity of skin symptoms. The score of IGA was 4, BSA was above 50%, EASI was 32 at the first visit. The level of serum total IgE was above 5,000 KU/L. We initiated treatment with Dupilumab, 600 mg at first dosage and then 300 mg every 2 weeks. After 8 weeks, the IGA score decreased to 1, BSA to 5% and EASI to 2.5. The Dermatology Life Quality Index (DLQI) score decreased from 16 at baseline to 5 at week 8. The serum total IgE level also decreased to 3,135 KU/L at week 12 (Table 1). In addition, the indicators related to NS, such as serum creatinine, serum albumin, urinary protein, urinary creatinine, and urine protein/creatinine ratio were within normal range, and the prednisone dosage decreased to 1.25 mg daily.

**Table 1:** Sera antibodies levels before and after Dupilumab treatment.

	**Before**				**After**			
	**IgG**	**IgM**	**IgA**	**IgE**	**IgG**	**IgM**	**IgA**	**IgE**
Patient 1#	7.7	1.51	1.83	>5,000	8.9	1.61	1.91	3,135
Patient 2#	11.7	0.79	2.05	492	11.1	0.84	1.88	223

*IgG, IgM, IgA: g/L; IgE: KU/L*.

**Figure 1:**
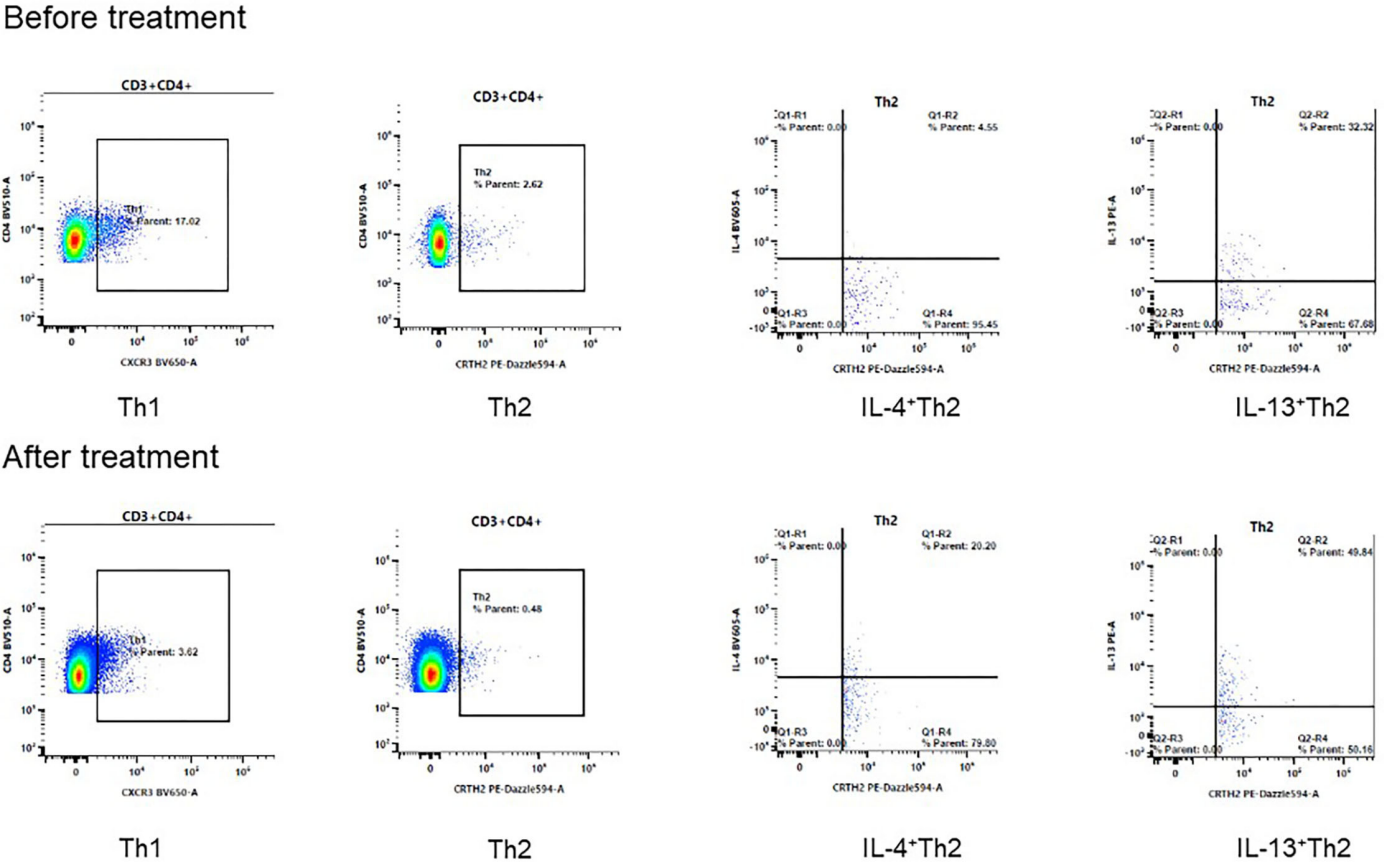
The changes of Th1 and Th2 cells after 8 weeks treatment of Dupilumab. The proportion of IL-4+Th2 cells increased from 4.55% to 20.20%, IL-13+Th2 cells increased from 32.32% to 49.84% after treatment in patient #2.

The second patient was a 13-year-old boy who also suffered from AD after birth. The manifestation of AD was similar to that of patient #1. Similarly, he didn't respond well to the treatment with topical glucocorticoids and oral antihistamine. He was diagnosed with AR and asthma when he was 3 years old, His father had AD and asthma history. He was diagnosed with NS 3 years ago and received oral prednisone and tacrolimus because of refractory proteinuria. When the patient presented to our department, he had a height of 155 cm, weight of 40 kg and the body mass index of 16.65 kg/m^2^. He received prednisone 10 mg every 2 days and Tacrolimus 2 mg/day to treat the NS comorbidity. His urinary protein test was negative at the dosage but turned to be positive when we tried to reduce the medication dosage. The score of IGA was 4, BSA was 40%, EASI was 21.4 at the first visit. The level of serum total IgE was 492 KU/L at baseline. We initiated treatment with Dupilumab, 600 mg at first dosage and then 300 mg every 3 weeks. After 8 weeks, the IGA score decreased to 1, BSA to 4%, EASI to 2.4 and total IgE to 223 KU/L (Table 1). The DLQI score was decreased from 17 at baseline to 4 at 8 weeks. The indicators related to NS were within normal range, and the prednisone dosage decreased to 7.5 mg every 2 days and Tacrolimus dosage decreased to 1.5 mg/day at week 8. The peripheral blood mononuclear cells were further collected for T cell subsets analysis. Interestingly, the proportion of IL-4 and IL-13 producing Th2 cells were increased after Dupilumab treatment (Figure 1, Table 2).

**Table 2:** Sera cytokines levels before and after Dupilumab treatment.

	**Before**				**After**			
	**IL-4**	**IL-13**	**IFN-γ**	**IL-10**	**IL-4**	**IL-13**	**IFN-γ**	**IL-10**
Patient 1#	13.5	15.2	59.4	13.4	15.4	17.1	59.7	12.6
Patient 2#	13.5	11.6	59.9	12.9	13.0	11.2	59.5	12.9

*IL-4, IL-13, IFN-γ, IL-10: pg/ml*.

The statutory guardians of patient #1 and patient #2 had given written informed consent to the publication of their case details. The study was conducted according to the Declaration of Helsinki.

## Discussion

Taken together, in the course of Dupilumab treatment, the AD symptoms of both patients were relieved significantly. Meanwhile, the dosages of prednisone or Tacrolimus for NS were also reduced, and no adverse reactions were found.

NS is an abnormal kidney condition marked by excretion of albumin in the urine and hypoalbuminemia due to altered permeability of the glomerular basement membranes (13). Although NS can affect people of any age, it's usually first diagnosed in children aged between 2 and 5 years old. Systemic steroids are the core treatment for NS with protocols based on seminal researches of the International Study of Kidney Disease in Children. However, there are still unmet needs for the management of NS in children. For example, most patients will relapse, with approximately half becoming frequently relapsed or steroid dependent, and it is well known that long term steroid use is associated with many side-effects including obesity, hypertension,Cushing syndrome, growth disorder, ocular complications, and osteoporosis (13).

Several studies showed that NS was closely related to allergic diseases (5, 14, 15) and type 2 inflammation (16–19). From an epidemiological aspect, Fanconi et al. (20) first linked atopy to NS in 1951. Many studies have shown that pediatric NS had a higher incidence of allergic diseases, including AD, allergic rhinitis, asthma, recurrent urticaria and hay fever (20). These patients also presented a higher serum IgE level (5, 15). Meanwhile, a large-population retrospective cohort study which enrolled 192,295 pediatric AD and 769,169 non-AD children showed the incidence of NS was significantly higher in the AD children compared with non-AD group, and the severity of AD also showed a positive correlation to NS incidence (5, 15). From pathogenesis aspect, there are also evidences implying the correlation between allergic diseases and NS. As the key mechanism of allergic diseases is the chronic inflammation mediated by T helper type-2 (Th2) cells (12, 21), allergic diseases are characterized by elevated levels of cytokines such as IL-4 and IL-13, which may play important roles in the pathogenesis of NS (16–19). Previous studies showed the levels of IL-4, IL-13 and IL-18 were significantly higher during the active stage of steroid sensitive nephrotic syndrome (SSNS) than remission stage and control group (17, 19). The percent of IL-13 producing CD3^+^ cells was significantly higher in the nephrotic relapse with steroids group compared with the nephrotic remission with steroids group (22). Animal studies also showed that IL-13 was involved in the pathogenesis of minimal-change nephrotic syndrome, and that the overexpression of IL-13 may lead to renal injury (23). There are emerging data suggested NS and AD might share similar pathogenesis (7). Thus, inhibition of type 2 inflammatory mediators such as IL-4/IL-13 may be a potentially effective therapy for NS (24).

Dupilumab is a human monoclonal antibody that can inhibit the signaling pathway induced by IL-4 and IL-13. It has shown convincing efficacy and good safety for the treatment of type 2 inflammatory diseases including AD, asthma, and chronic rhinosinusitis with nasal polyposis (24). Currently, there are no reports of the efficacy and safety of Dupilumab in patients with NS. Based on the epidemiological and basic research data of AD and NS, we hypothesize that Dupilumab may be a potential therapeutic medication for NS patients, at least not a contraindication. In addition, some studies suggested that Th2 and related cytokines IL-4 and IL-13 also involved in other kidney diseases, such as idiopathic focal segmental glomerulosclerosis and correlated with lower cortico-resistance (25). Theoretically, Dupilumab might be an alternative option if the patients are reluctant or intolerant to long-term corticosteroid treatment.

Our hypothesis was preliminarily validated in the two cases which both showed good control of NS and less systemic steroids and/or immunosuppressive agents use during the Dupilumab treatment period, accompanied by significant relief of AD symptoms. The CD4^+^T cell subsets analysis in one patient showed the proportion of IL-4 and IL-13 producing Th2 cells were increased after Dupilumab treatment. In contrast to the decreased IgE level, we hypothesize the increased IL-4 and IL-13 producing Th2 cells may be a response to suppressed IL-4 and IL-13 functions. However, serum IL-4 and IL-13 levels of the two patients didn't change significantly after Dupilumab treatment. We also found the other antibodies such as IgG/IgA/IgM and cytokines such as IFN-γ/IL-10 were not changed. Thus, more data is needed to validate our findings and elucidate the exact mechanisms of Dupilumab in NS. We suggest prospective pilot studies and randomized controlled trials could be carried out to validate the efficacy and safety of Dupilumab in the treatment of NS patients.

## Data Availability Statement

The raw data supporting the conclusions of this article will be made available by the authors, without undue reservation.

## Ethics Statement

Written informed consent was obtained from the participants' statutory guardian for the publication of any potentially identifiable images or data included in this article.

## Author Contributions

R-FZ and L-RQ conceived the idea and prepared and revised the manuscript. Y-QY and HC collected the data. All authors contributed to the article and approved the submitted version.

## Conflict of Interest

The authors declare that the research was conducted in the absence of any commercial or financial relationships that could be construed as a potential conflict of interest.

## Publisher's Note

All claims expressed in this article are solely those of the authors and do not necessarily represent those of their affiliated organizations, or those of the publisher, the editors and the reviewers. Any product that may be evaluated in this article, or claim that may be made by its manufacturer, is not guaranteed or endorsed by the publisher.
